# Eureka

**Published:** 1888-06

**Authors:** 


### EUREKA !

It has long been known that the world is full of microbes; that all sickness and disease are caused by them. The most important thing to be discovered was how to get rid of them; how we can kill them or prevent them from doing us harm.

What has been needed is something that would kill the microbes and not the patient.

An ingenious citizen of Texas announces to all mankind that he has found "that thing" sure and certain. He says: "To remove all doubt that I have a genuine microbe killer or destroyer, I refer to the cures that have been made by the use of this preparation. I can prove it as a fact, that most of the persons I have cured I have never seen; neither did I know, in many instances, what their disease was, and, although their maladies were different and often complicated, yet the continued use of the Microbe Killer cleaned them out and cured them. What I have already done I feel myself perfectly competent to explain, and why I claim the Microbe Killer will cure all diseases. A good many persons are well up in talking and writing, and yet are not able to help themselves.

"See that little bit of sickness with which the Crown Prince of Germany is suffering. Is he not a poor man in spite of all his power and wealth? The doctors say it will kill him, and yet thirty dollars worth of Microbe Killer would set him all right again. I would not charge him even that much. But, alas, there are some smart men over there who cannot believe that such a thing as a Microbe Killer can be found, and especially in Texas. Texas is the very country for inventions, and this is the greatest invention or discovery that has ever been made, because it preserves life, and when we lose our lives we lose all that we have."

